# Partners in Parenting: A Multi-Level Web-Based Approach to Support Parents in Prevention and Early Intervention for Adolescent Depression and Anxiety

**DOI:** 10.2196/mental.8492

**Published:** 2017-12-19

**Authors:** Marie BH Yap, Katherine A Lawrence, Ronald M Rapee, Mairead C Cardamone-Breen, Jacqueline Green, Anthony F Jorm

**Affiliations:** ^1^ Monash Institute of Cognitive and Clinical Neurosciences School of Psychological Sciences Monash University Clayton Australia; ^2^ Melbourne School of Population and Global Health University of Melbourne Melbourne Australia; ^3^ Centre for Emotional Health Macquarie University New South Wales Australia

**Keywords:** family, tailored, internet, mental health, preventive health services

## Abstract

Depression and anxiety disorders in young people are a global health concern. Various risk and protective factors for these disorders are potentially modifiable by parents, underscoring the important role parents play in reducing the risk and impact of these disorders in their adolescent children. However, cost-effective, evidence-based interventions for parents that can be widely disseminated are lacking. In this paper, we propose a multi-level public health approach involving a Web-based parenting intervention, Partners in Parenting (PIP). We describe the components of the Web-based intervention and how each component was developed. Development of the intervention was guided by principles of the persuasive systems design model to maximize parental engagement and adherence. A consumer-engagement approach was used, including consultation with parents and adolescents about the content and presentation of the intervention. The PIP intervention can be used at varying levels of intensity to tailor to the different needs of parents across the population. Challenges and opportunities for the use of the intervention are discussed. The PIP Web-based intervention was developed to address the dearth of evidence-based resources to support parents in their important role in their adolescents’ mental health. The proposed public health approach utilizes this intervention at varying levels of intensity based on parents’ needs. Evaluation of each separate level of the model is ongoing. Further evaluation of the whole approach is required to assess the utility of the intervention as a public health approach, as well as its broader effects on adolescent functioning and socioeconomic outcomes.

## Introduction

### Overview

Depression and anxiety disorders are the largest contributors to disease burden in young people globally [1]. Research evidence highlights that parents have an important role in reducing the risk and impact of these disorders in their adolescents; however, cost-effective, evidence-based interventions for parents that can be widely disseminated are lacking. In this paper, we propose a multi-level public health approach involving a Web-based parenting intervention to address this dearth of resources for parents across all levels of this continuum [2].

### Depression and Anxiety Disorders in Youth Are a Global Health Concern

In young people aged between 13 to 17 years, lifetime prevalence rates of depression and anxiety disorders are 18% and 38%, respectively [3]. Early onset disorders, especially if untreated, tend to become chronic or relapsing, increase suicide risk, and forecast a wide range of psychosocial and vocational impairments [4-6]. Although intervention efforts for these disorders continue to progress, and rates of professional help seeking have increased [7], a large proportion of the burden of disease is still unavertable even with optimal treatment [8]. There is, hence, a strong need for an effective, integrated approach to reduce the prevalence and impact of these disorders, especially for young people. As the incidence of these disorders peaks during adolescence [9,10], adolescence is a particularly opportune time to target prevention and early intervention (referring to treatment and maintenance early in the course of disorder).

### Parents Have an Important Role in Prevention and Early Intervention

There are various reasons why the family, particularly parents, is a strategic setting for targeting prevention and early intervention for youth depression and anxiety (also known as internalizing) disorders. First, young people see their family, especially their parents, as important in their lives, especially when it comes to their own mental health. Various national surveys have found that parents are the most commonly mentioned source of help young people would turn to if and when they have mental health difficulties [11,12]. Second, parents are intrinsically motivated to take action for their child’s well-being and may possess the wisdom and life experience to help them appreciate the value of prevention and early intervention [13]. Third, most adolescents still live with their parents (or at least one parent), and this proximity affords parents the opportunities to notice significant changes in their child’s mental health and behavior. As argued by proponents of family process [14] and family system [15] models, this proximity underscores the importance of parents in the development and maintenance of youth internalizing problems. Fourth, international policies and action plans related to mental health have recognized the importance of upskilling parents for the goal of prevention and promotion of child and youth mental and emotional well-being [16-19].

Finally, there is now robust evidence delineating risk and protective factors for adolescent anxiety and depressive disorders [20,21]. Importantly, some of these factors are within parents’ control or influence and are potentially modifiable [22]. These include factors that involve the family system (eg, interparental conflict [23]), can be detected early by parents (eg, behaviorally inhibited temperament [20]), or are directly socialized or modeled by parents (eg, parental responses to child emotions [24]). However, findings from a national survey of Australian parents revealed that parents’ knowledge about their role in reducing risk of depression in adolescents is less than optimal [25], highlighting a need to equip parents through more effective translation of evidence into preventive resources.

Moreover, a substantial body of research has demonstrated the various ways in which parenting behaviors may inadvertently maintain or exacerbate depression and anxiety disorders in young people [14,15,26,27]. For instance, as proposed by reciprocal relationship models, adolescent anxiety may elicit overprotective responses from parents, which in turn reinforces and maintains adolescent anxiety [28]. Parental modeling of anxiety [14] or maladaptive strategies to manage their own emotions [29] may also contribute to the maintenance of adolescent internalizing problems.

The rest of this paper presents the rationale for developing Partners in Parenting (PIP), an individually tailored Web-based intervention for parents of adolescents. We then describe the intervention development process and explain how the various components were designed to facilitate the proposed multi-level approach to empower parents to reduce the risk and impact of depression and anxiety disorders in their adolescent children.

### Rationale for Developing the Partners in Parenting Intervention

Below, we describe the three key reasons that motivated the development of the PIP and the proposed multi-level approach.

#### Need to Involve Parents Across the Mental Health Intervention Continuum

Existing research evidence demonstrates the value of involving parents across the mental health intervention continuum, which includes prevention (universal, selective, and indicated), treatment (case identification and standard treatment for known disorders), and maintenance (strategies to reduce relapse and recurrence, and the disability associated with the disorder) [2].

Preventive parenting interventions can be *universal* (ie, delivered to all parents regardless of risk), *selective* (targeting parents whose children have known risk factors), or *indicated* (targeting parents whose children show signs or symptoms of emerging disorders) [2]. Although universal programs tend to have smaller effects than selective or indicated programs at the level of the individual, they can have a great public health impact because they reach a larger proportion of the population and have the potential to shift the population mean levels of depression and anxiety symptoms [30]. Notably, in a recent systematic review and meta-analysis of preventive parenting interventions (where most of the intervention was with the parent, rather than targeting primarily the child or involving the whole family), there was no evidence that the type of prevention (universal, selective, or indicated) moderated intervention effects [31]. When trying to engage parents in prevention of youth mental health problems, universal approaches can increase acceptability because they minimize the perceived stigma that some parents fear would be attached to themselves as a “bad” parent or to their child as having problems needing intervention [32]. On the other hand, according to the widely used Health Belief Model [33]—which explains why individuals engage in health-related behaviors—parents whose child has known risk factors (selective prevention) or early signs of difficulties (indicated prevention) may be more motivated to participate in preventive parenting programs because of heightened “perceived susceptibility” of their child.

Parents have an important role in facilitating case identification and professional help-seeking for adolescents. Parents are often the first to detect changes in their child’s mental health and serve as an important conduit to adolescents engaging in appropriate treatment [34]. Given the evidence for parenting-related risk, protective, and maintenance factors in adolescent internalizing disorders [14,15,31], parents also have an important role in the maintenance component of the mental health intervention continuum.

Hence, we propose a multi-level public health approach involving the PIP Web-based intervention that incorporates universal, selective, and indicated prevention components, as well as treatment and maintenance components to maximize the strengths of all components to meet the needs and preferences of different families.

#### Prevention and Early Intervention Programs Fail to Adequately Involve Parents

One important limitation of existing prevention and early intervention programs for adolescent internalizing disorders is the inadequate level and nature of parental involvement. Specifically, whereas some interventions include a parent component, this usually involves teaching parents what their child is being taught, rather than targeting modifiable parenting risk, protective, or maintenance factors [14,23]. In particular, given that most existing treatments utilize cognitive behavioral therapy (CBT) approaches that primarily target cognitions and behaviors at the individual (adolescent) level, parental involvement often takes the form of supporting the child’s implementation of strategies taught in session [35,36]. In many existing interventions, where a parent component exists, it tends to comprise a small proportion of the intervention, with the majority of the intervention targeting the young person [35-40].

In contrast to the increasing number of interventions targeting young people primarily [35,36,38-40], the aforementioned review of preventive parenting interventions found only three out of 51 interventions that were designed for parents of adolescents [31]. Notably, whereas preventive parenting interventions were found to have beneficial effects on the child’s internalizing outcomes lasting up to 11 years post intervention [31], the evidence base for preventive interventions targeting young people directly suggests that intervention effects may last less than 2 years [38-40]. These findings underscore the need to provide parents of adolescents with more evidence-based parenting support, to reduce their adolescent’s risk of internalizing disorders.

The dearth of interventions for adolescent depression and anxiety disorders that directly target parenting factors [35,36] stands in stark contrast to the myriad of family-based intervention programs for externalizing or substance use disorders in young people [15]. This lag in research translation is particularly notable given that meta-analyses of parenting factors have found comparable effect sizes for associations with youth externalizing problems (up to 6% [41], or up to 11% for delinquency [42]), substance use problems (eg, alcohol misuse, up to 7% [43]), and internalizing problems (up to 16% [23]). Moreover, evidence to date indicates no difference in treatment outcome between individual, group, and family or parental formats of CBT approaches for child and adolescent anxiety disorders [44].

Efforts to translate research evidence on the role of parenting in the maintenance of adolescent depression and anxiety may be deterred by the equivocal evidence to date regarding whether parental involvement in adolescent treatment enhances treatment effects [15,45]. However, given the dearth of treatment interventions for adolescent internalizing disorders that target parenting-related maintenance factors, it remains to be ascertained whether such an intervention will indeed enhance treatment effects. Nonetheless, professional help-seeking for adolescent depression or anxiety is often facilitated by parents [34] who want to help but do not always know how [15] and may inadvertently contribute to the maintenance of their child’s difficulties [27]. Hence, the PIP intervention was developed to address the need for an intervention that targets evidence-based parenting-related risk, protective, and maintenance factors and empowers parents to reduce the risk and impact of internalizing problems in their adolescent children.

#### Potential of the Web-Based Platform to Address Some Limitations of Existing Parenting Interventions

Another limitation of existing parenting interventions is that many are not well-used, even when available, because of barriers such as scheduling difficulties or privacy concerns [46]. With the increasing reach of the Internet, the use of Web-based media has been recommended as one key way to increase participation rates in preventive interventions [47]. For example, in Australia, the 2016 national census found that 97% of households with children younger than 15 years have Internet access [48]. However, based on the recent systematic review [31] and a search of major clinical trial registries, there is currently no widely accessible, tailored Web-based parenting intervention for prevention or early intervention for adolescent depression and anxiety disorders. Yet, Web-based interventions hold great promise because they have the potential to overcome the aforementioned barriers of existing face-to-face programs because of their anonymity, flexibility, and accessibility. Furthermore, the computerized delivery of a well-designed and well-maintained program guarantees implementation fidelity [49]. A Web-based parenting program also complements the use of the Internet as a popular source of information on mental health and parenting [7,50]. Moreover, a recent Web-based survey suggests that the majority of parents would find such a program useful [51].

Web-based interventions have now demonstrated effectiveness [49] and cost-effectiveness [52] for the treatment of depression and anxiety disorders. Promising evidence is also emerging for online prevention programs targeting young people directly [53], as well as parents of younger children [54,55]. The potential efficacy of Web-based prevention programs that target parents of adolescents remains largely untapped, but such programs would comprise a promising public health approach to preventing adolescent depression and anxiety that is potentially lower in cost per individual than existing programs [56].

An important limitation of existing prevention and treatment interventions for adolescent internalizing disorders is that they only focus on one or a few parenting risk, protective, or maintenance factors for adolescent depression and anxiety [14,15,31,45]. This narrow focus approach means that programs may not adequately address the range of modifiable parenting factors for adolescent depression and anxiety that are relevant for each parent or family. The capacity of digital technology to automatically tailor a Web-based intervention to each user offers a potential solution to this limitation. Automated tailoring is beneficial when it involves screening each parent across all evidence-based risk, protective, and maintenance factors to ensure a more thorough coverage of areas that may be important to target in the intervention. In doing so, the program has greater breadth without imposing unnecessary burden on parents (because of the inclusion of less-relevant topics). Importantly, a tailored Web-based intervention provides some personalization of the program for the parent without requiring the costly involvement of trained professionals, hence increasing the intervention’s perceived relevance [57], effectiveness [58], and potential for scalability and sustainability [57].

## The Partners in Parenting Intervention

The PIP intervention comprises three components: (1) a parenting scale that assesses the parent’s current parenting practices and beliefs against the recommendations of the parenting guidelines; (2) an automatically generated, individually tailored feedback report based on each parent’s responses to the scale; and (3) a set of interactive Web-based modules to support parental behavior change.

To access PIP, parents register by creating an account using their email address and a self-selected password and providing basic demographic information about themselves and their child. To personalize the intervention to each parent, parents are asked to identify one target child to focus on when completing the intervention. All components of PIP are then personalized with the child’s name and gender and the parent’s name. Parents then complete the parenting scale to receive their tailored feedback report, before reviewing the selection of modules recommended specifically for them, alongside other modules (out of the nine) that are also available but were not recommended for them (because they were already considered concordant with the guidelines’ recommendations in those areas of parenting). At this point, parents can apply their own preferences by accepting or deselecting recommended modules and selecting any additional modules that were not recommended before locking in their selection and starting their personalized program. One module is unlocked every 7 days, in a predetermined order (because each subsequent module is designed to build on the content of preceding modules), until the parent has completed all of the modules in their program. The 7-day interval encourages parents to focus on achieving the goal they had set from their most recently completed module before proceeding to the next module. Parents receive an automated email informing them that their next module is available and reminding them of the goal(s) they had previously selected but not yet marked as achieved on their personalized dashboard. Each module takes 15 to 25 min to complete depending on the module and the way parents choose to engage with it. After completing all of their modules, parents gain access to all modules, including those they had not initially selected (see Multimedia Appendix 1 for screenshots).

## Development of the Partners in Parenting Intervention

### Overview

The development of PIP involved three phases that were modeled after the related Parenting Strategies intervention to prevent adolescent alcohol misuse [59]. The Center for eHealth Research (CeHRes) roadmap for the development of electronic health (eHealth) technologies [60] guided the process of user-centered design. Specifically, the first two phases comprised a research translation process to develop a set of guidelines that represent the range of risk and protective factors to target in the intervention (akin to CeHRes Contextual Inquiry activities—identifying user needs and possible solutions). Phase 3 was guided by the consumer-engagement approach for developing parenting programs (CeHRes Value Specification—determining what users value) [61], and the intervention’s Web-based technological features were designed to fulfill the principles of the Persuasive Systems Design (PSD) model (CeHRes Design—iterative process of building, testing, and refining prototypes and incorporating persuasive techniques) [62]. Considerations about the PIP implementation model were inherent throughout the development process (CeHRes Operationalization—introduction, adoption, and employment of the technology in practice).

### Phase 1. Identifying Parental Factors to Target in the Intervention

To identify the range of modifiable parental factors to target in the intervention, the first phase involved a comprehensive systematic review and meta-analysis of risk and protective factors for adolescent depression and anxiety disorders that parents can potentially modify or influence [23]. Synthesizing longitudinal, retrospective, and cross-sectional evidence, the review identified a sound evidence base for three protective parental factors for depression (warmth, autonomy granting, and monitoring), and one for anxiety (warmth). In addition, three risk factors for both outcomes were also identified: interparental conflict, overinvolvement, and aversiveness [23].

### Phase 2. Translating the Research Evidence Into Actionable Strategies

To translate this evidence base into actionable strategies, we employed the Delphi method to develop a set of expert consensus guidelines [63]. The Delphi method is a systematic way to determine expert consensus about questions that cannot be appropriately or adequately addressed using experimental or epidemiological methods [64]. This phase involved a systematic literature search of both academic and lay information, which identified 402 unique recommendations for parents to reduce the risk of depression or anxiety in their adolescent. An international panel of 23 clinical and research experts independently rated these recommendations over three survey rounds. Panel members were provided with brief summaries of the evidence from the systematic review of research evidence [23] to consider when rating the items.

The resulting guidelines *, How to prevent depression and clinical anxiety in your teenager: Strategies for parents* (henceforth “the Guidelines”; [65]) presents 190 parenting strategies that were endorsed by ≥90% (21/23) of experts as important or essential for the prevention of adolescent depression and anxiety disorders. These strategies were thematically organized under 11 subheadings, as shown in Table 1.

The Guidelines [65] represent evidence-based and expert-endorsed strategies that parents can use to reduce their adolescent’s risk of depression and anxiety problems. A recent study evaluating user perceptions of the Guidelines indicated high levels of satisfaction, and the majority of users endorsed the potential value of Web-based parenting interventions based on the guidelines. Most parent users also reported attempting to make changes in their parenting as a result of reading the Guidelines [51]. Albeit a preliminary and uncontrolled evaluation study, these findings suggest the utility of the Guidelines as a basic, universal prevention strategy for parents of adolescents.

### Phase 3. Developing the Web-Based Intervention

#### The Persuasive Systems Design Model

To support parents in the implementation of the Guidelines, and to individually tailor the Guidelines’ recommendations to each parent, phase 3 involved developing the three aforementioned components: (1) a self-assessment parenting scale, (2) a tailored feedback report, and (3) a set of interactive Web-based modules.

Design of the Web-based components of PIP was guided by the PSD model [62] that proposes to purposefully use technology to influence behavior change. In particular, the key features of PIP were designed to fulfill the principles of the PSD model in the primary task, dialogue, and system credibility categories (see Multimedia Appendix 2) [62].

#### Intervention Components

First, we developed a criterion-referenced parenting scale, called the Parenting to Reduce Adolescent Depression and Anxiety Scale (PRADAS), which assesses parents’ concordance with the nine domains of parenting addressed in the nine subheadings of the Guidelines (the “criterion”; see [66] for more details). The PRADAS represents the screening assessment that facilitates the tailoring of the intervention to each parent [57].

Next, we wrote automated feedback messages for all possible combinations of responses to the 79 items in the PRADAS. This involved creating a scoring system and feedback flowchart linking the response options for each item to the appropriate feedback message based on the predetermined scoring algorithm. Feedback messages highlight the parent’s parenting strengths and provide specific strategies to further improve their parenting, to adhere more closely to the recommendations of the Guidelines. Feedback messages are intentionally written to be brief, with the aim of motivating behavior change by identifying areas to change and providing specific means for action (PSD tunneling principle, [62]). The recommended behavior change is then supported by corresponding modules (see below) that are specifically recommended for each parent to build on the strategies presented in the personalized feedback. The tailoring of every feedback message increases the perceived relevance of the intervention and allows the intervention to cover the range of factors that represent areas for improvement for each parent. The PRADAS content and feedback messages were initially drafted by a postgraduate student with graduate qualifications in psychology (MCB) and evaluated by the research team (comprising MCB, MBHY, AFJ, and KAL) to ensure their fidelity to the Guidelines.

Finally, the development of the interactive modules first involved a mapping of topics to the nine domains of parenting addressed in the Guidelines (see Table 1). Modules feature full colored illustrations, interactive activities, real-life vignettes, audio clips, troubleshooting tips, goal setting exercises, and an end-of-module quiz with immediate feedback to consolidate learning of each module’s key messages. Features of the modules were selected to fulfill PSD principles, and as part of the consumer-engagement approach [61], taken to develop both PIP and the earlier alcohol misuse prevention intervention [59]. Module content was based on the Guidelines but drew on other relevant evidence-based content as required. A psychologist undertaking postgraduate research (JMG) drafted the initial modules, which were then reviewed and revised through meetings involving the research team (comprising JMG, MBHY, AFJ, and KAL). Module content was evaluated to ensure its consistency and fidelity with the Guidelines, as well as other relevant best practice and credible resources.

Attention was paid to ensure that all components of the intervention were optimized to engage parent users, following the PSD principles as far as possible (as outlined in Multimedia Appendix 2). As part of a consumer-engagement approach to developing the intervention [61], we also consulted with parent and adolescent stakeholders to ensure that the various components of the intervention fulfilled the PSD principles as intended and were acceptable to target end users (see below).

#### Stakeholder Consultations—Parents

We recruited a reference group of 22 parents with adolescent children (aged 11-18 years) through staff e-newsletters at Monash University and the University of Melbourne, local high schools, and via online networks. Participants were mostly mothers (86.4%, 19/22), in the age range of 45 to 59 years, married or de facto, employed, Australian-born, English-speaking, and highly educated (at least an undergraduate qualification) and had 2 or 3 children. Parents attended one of three repeated 2-hour workshops (n=7 or 8 per workshop) where drafts of the PRADAS, feedback messages, and one module prototype (drafted as a Microsoft PowerPoint presentation) were presented for discussion. Parents were consulted on the language used in the PRADAS and feedback messages, and the logic, relevance, and usefulness of the feedback messages. They also provided feedback and input into the degree of interactivity and the tone and amount of content in the modules. Parents provided specific suggestions for rewording instructions and messages that could be misinterpreted or trigger unintended negative reactions from parents. Wherever possible, we incorporated parents’ feedback into all components of the intervention.

**Table 1:** Guidelines topics, corresponding subsections of the parenting scale and personalized feedback report, title of interactive modules, outline of content, and rationale for their inclusion.

Guidelines topic	Corresponding subsection of the parenting scale and feedback report	Title of interactive module	Outline of content	Rationale for inclusion
You can reduce your child’s risk of developing depression and clinical anxiety	N/A^a^; Not included in parenting scale or feedback report	N/A; No module on this topic	Psychoeducation about the role of parents in the prevention of adolescent depression and anxiety	Endorsed by experts
Establish and maintain a good relationship with your teenager	Your relationship with your teenager	Connect	Acknowledges the challenge of connecting with adolescent children, and provides specific tips on how to do this	Sound research evidence that parental “warmth” is protective against both anxiety and depression; endorsed by experts
Be involved and support increasing autonomy	Your involvement in your teenager’s life	Nurture roots and inspire wings	Helps parents establish the important balance between staying involved and interested in their adolescent’s life, while encouraging increasing age-appropriate autonomy	Sound research evidence that overinvolvement is a risk factor for depression, and autonomy granting and monitoring are protective factors; endorsed by experts
Encourage supportive relationships	Your teenager’s relationships with others	Good friends, supportive relationships	Provides strategies for parents to support their adolescent’s social skills development	Emerging evidence of parental encouragement of sociability is associated with less adolescent anxiety; endorsed by experts
Establish family rules and consequences	Your family rules	Raising good kids into great adults: establishing family rules	Highlights the importance of consistent and clear boundaries for adolescent behaviors, and provides specific strategies to establish these	Emerging evidence of the association between inconsistent discipline and depression; endorsed by experts
Minimize conflict in the home	Your home environment	Calm versus conflict	Addresses the need for adaptive conflict management between parents and between parent and adolescent, and provides specific strategies to do these	Sound evidence that interparental conflict and aversiveness (including parent-adolescent conflict) are risk factors for both depression and anxiety; endorsed by experts
Encourage good health habits	Health habits	Good health habits for good mental health	Provides strategies to help parents encourage good health habits in their adolescent, including a healthy diet, physical activity, good sleep habits, and abstinence from alcohol and drugs	Endorsed by experts; evidence that these health habits are associated with risk for depression and anxiety
Help your teenager to deal with problems	Dealing with problems in your teenager’s life	Partners in problem solving	Provides strategies for parents to help their adolescent develop good problem solving and stress management skills	Endorsed by experts
Help your teenager to deal with anxiety	Coping with anxiety	From surviving to thriving: helping your teenager deal with anxiety	Provides strategies for parents to help their adolescent manage their everyday anxiety	Sound evidence that overprotective, anxious parenting is associated with both anxiety and depression in adolescents; endorsed by experts
Encourage professional help seeking when needed	Getting help when needed	When things aren’t okay: getting professional help	Helps parents understand what depression and anxiety problems can look like in adolescents, and what they can do if their adolescent is or becomes unwell	Endorsed by experts; evidence that parents are important conduits to young people seeking professional help for mental health problems
Don’t blame yourself	Don’t blame yourself (not included in parenting scale, included in feedback report for all parents)	N/A; No module on this topic	Aims to dispel guilt or self-blame in parents	Endorsed by experts

^a^N/A: not applicable.

#### Stakeholder Consultations—Adolescents

Finally, to ensure that the suggested strategies recommended to parents in the intervention were acceptable and relevant to adolescents, we consulted with two focus groups of adolescents in the age range of 12 to 15 years. Adolescents were recruited through two local schools that differed on ethnic and sociodemographic characteristics, and focus group consultations were conducted in school classrooms. Consulting with adolescents of different ages and in different schools enabled us to capture some developmental, ethnic, and sociodemographic variations in adolescent views. Adolescents were presented with some of the parenting strategies recommended for parents in PIP (eg, show interest in your adolescent’s life and spend regular one-on-one time together) and provided feedback about some ways in which the strategies could be implemented in an acceptable way with contemporary adolescents. Adolescents provided specific ideas and suggestions that were incorporated into the content of various modules, including activities they enjoy doing with their parents and ways their parents could show them affection. These consultations also informed the scripts for adolescent audio clips included in some modules, where adolescents talked about topical issues such as how they feel when their parents argue, and how parents could help when they (the adolescents) get “stressed out.”

The PIP intervention development was completed in May 2015. We are evaluating the effects of the intervention via two randomized controlled trials (RCTs) that have been registered with the Australian New Zealand Clinical Trials Registry (Trial IDs ACTRN12615000247572 and ACTRN12615000328572).

## Proposed Uses of the PIP Intervention: A Multi-Level Public Health Approach

### Overview

**Figure 1:**
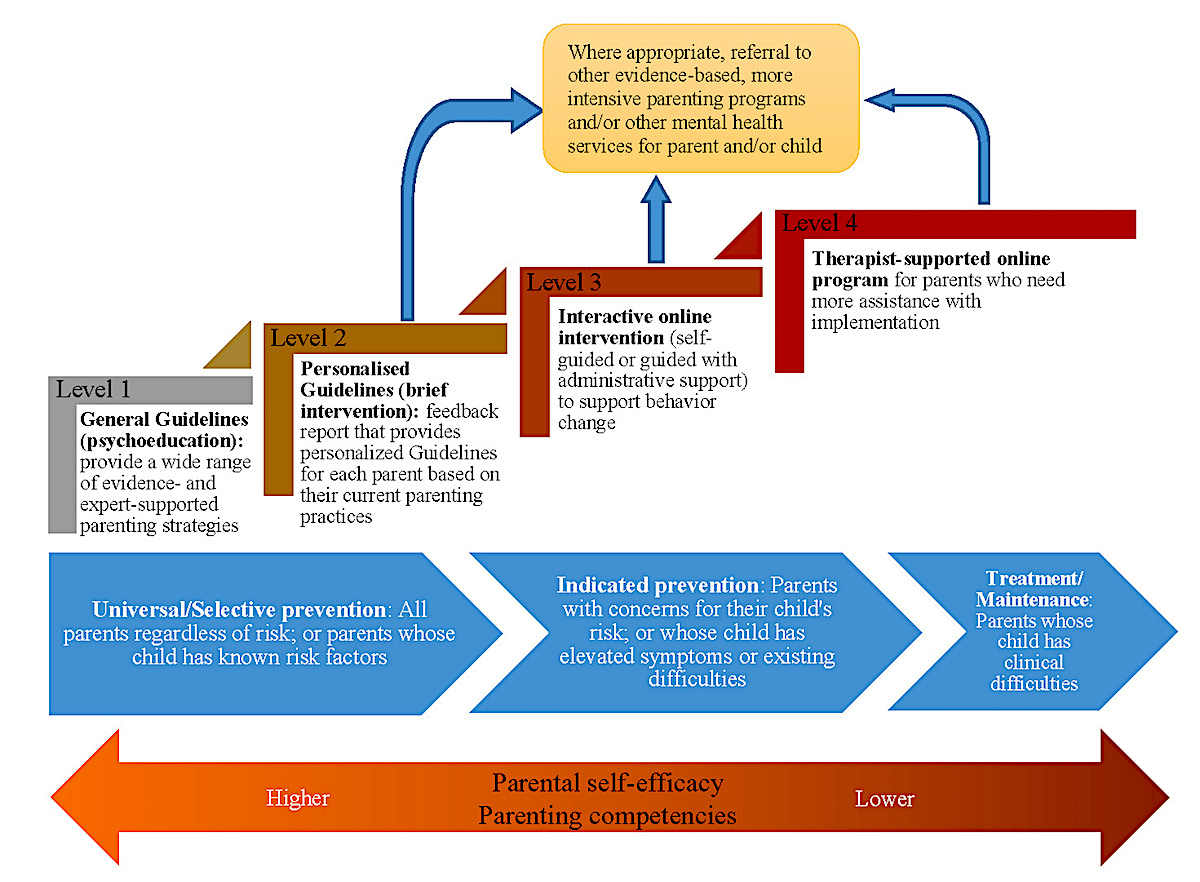
A multi-level public health approach to support parents.

The PIP intervention was designed for implementation as a multi-level public health intervention to empower parents to support their child’s mental health across all levels of the mental health intervention continuum [2]. Figure 1 depicts a model of the proposed that involves different PIP components in varying degrees of intensity (or levels). We propose that the level of PIP required will be related to the level of risk and extent of current difficulties in the child [2], as well as the parent’s self-efficacy (confidence about their ability to parent successfully) and parenting competencies or skills [67,68].

### Level 1: General Guidelines

Level 1 is the minimal intervention and constitutes a general parent-education initiative across the community. Parents can choose to consider and apply any of the Guidelines’ recommendations as and when they deem fit. Given the evidence base [23] and expert endorsement [63] supporting these recommendations, we postulate that when parents apply these strategies, they are taking preventive actions that are likely to benefit their child’s mental health. Given preliminary evidence that accessing these guidelines was sufficient to prompt some behavior change in parents [51], these guidelines represent a promising minimal-cost universal prevention strategy for parents of adolescents. This minimal intervention is likely to be sufficient for parents who are highly motivated, educated, and have higher parental self-efficacy and parenting competence and whose child is generally functioning well (ie, no known risk or current concern). The Guidelines can serve as a benchmark for parents, providing reminders of strategies to maintain, increase, or reduce, a toolkit to draw from as required, as well as an assurance that they are “on the right track” [51].

### Level 2: Personalized Guidelines (Brief Intervention)

Each subsequent level in the model represents increasing intensity of support and intervention for parents. Level 2 requires parents to first complete a self-assessment parenting scale (the PRADAS) to receive their personalized feedback report. This level is likely to suit a similar group of parents as level 1 but who prefer a tailored approach. Level 2 can also serve as a prompt for some parents to take further action, if required, to seek further support to improve their parenting practices. Parents with lower parental self-efficacy may find the level of support provided by a once-off brief intervention such as the feedback report insufficient and thus, be prompted to complete the interactive Web-based program (next level up) and/or seek other resources or services including mental health services for themselves and/or their child. To facilitate this, the feedback report includes links to other online resources, including an online screening tool (the Strengths and Difficulties Questionnaire; [69]) for parents who are concerned about their child’s mental health.

### Level 3: Interactive Online Intervention

At level 3, parents receive both the tailored feedback report and are recommended specific modules to provide additional support to implement the strategies highlighted in their feedback report. Drawing heavily from PSD principles, the intervention is designed to maximize adherence as a self-guided program [70], with automated email reminders and prompts to guide parents through their program to completion. We expect that parents who are motivated to improve their parenting and have moderate levels of parental self-efficacy would successfully complete their program on their own. However, evidence to date indicates that having some form of human support, be it administrative or therapeutic, enhances adherence to Web-based interventions (ie, completing the program as designed) and in turn, improves outcomes [71]. Hence, to maximize the potential benefits and cost-effectiveness of PIP, it may need to be delivered with at least administrative support, following a specified protocol (eg, a standard script with specific prompts to encourage progress through the Web-based program). It is pertinent that personnel delivering such administrative support have comprehensive training and ongoing supervision in the requisite skills to communicate with parents in a supportive and nonjudgmental manner and are equipped with referral information to additional support services (including level 4 of PIP) as required. Given the greater intensity of intervention that parents need to commit to, level 3 is more likely to appeal to parents who have some cause for concern (*selective prevention*; eg, lower parental self-efficacy during the child’s developmental transition into adolescence) or have existing concerns for their child’s mood or behavior (*indicated prevention*) [2]. 

However, a recent review (Finan SJ et al, 2017, unpublished data) found that although higher child mental health symptoms may be associated with initial engagement (eg, enrolment) in preventive parenting programs, this does not increase attendance or reduce the likelihood of parents dropping out of programs [72]. In a Web-based intervention such as PIP, it may be possible to partially ameliorate this challenge by providing the personal administrative-support contact [71].

### Level 4: Therapist-Supported Online Intervention

At level 4, parents receive not only all components of the PIP Web-based intervention but also the support of a trained therapist to coach them in implementing the strategies recommended in the PIP program. According to the Health Belief Model [33], this form of human support can act as a “cue to action” and help to increase intervention adherence through accountability to a coach who is perceived to be trustworthy, benevolent, and having expertise [73]. This additional level of support is particularly important when the child is already experiencing clinical-level difficulties because of their association with heightened stress in the family and reduced parental self-efficacy and parenting competence [67,68]. As noted earlier, there is a dearth of evidence-based supportive resources or services for parents of adolescents in the clinical setting [74]. Due to the increasing individuation from parents that emerges during adolescence [75] and a corresponding clinical imperative to promote independence and self-reliance in adolescents, parents are commonly less involved in treatment with their adolescent than they are with younger children. Inevitably, this can leave concerned parents feeling excluded from their child’s care, disempowered and helpless about how they can best manage their child’s condition outside the clinic, and frustrated when they are unable to access support for themselves from the child’s clinician [51,76]. Various systemic factors may also contribute to this, including the funding structure of public mental health services being directed at individuals rather than families, the professional competencies of youth mental health clinicians being limited to working with individual clients rather than the family system, and a largely overloaded and reactive mental health system. Within this context, the PIP intervention can be adapted for use to meet the critical gap in support services for parent caregivers of young people with internalizing disorders. Parents can access PIP with a separate PIP therapist-coach, who will, with the parent’s consent, communicate with the child’s clinician about the support the parent is getting from PIP, with the goal of enhancing their child’s treatment. Alternatively, youth mental health clinicians can be trained in PIP content as part of their professional and specialist training and development, which will enable them to provide coaching to parents who access PIP in their own time, in addition to the individual work done with the adolescent, as well as some family sessions. The PIP therapist-coach can capitalize on the automated tailoring features of PIP by using their parent client’s PRADAS responses and feedback report as a basis for discussion during coaching sessions. Evidence to date suggests that such an approach is likely to facilitate the young person’s recovery [14,74], support parents in their caregiver role, and increase adherence to treatment [76] without imposing significant added burden on the already overloaded treatment services because of PIP’s Web-based delivery.

### Criteria for Stepping Up

Within the proposed model, stepping up is based on one or both of the following criteria: (1) automated recommendation of the tailored program based on parents’ responses to a self-assessment of their current parenting (parenting competencies as assessed by the PRADAS), parental self-efficacy, or their child’s current symptoms and/or (2) parents’ personal preference, which can override the program’s recommendation. Referral to other evidence-based, more intensive parenting programs can occur at any point throughout the model for parents who want programs with a different delivery mode, increased support (therapist or nontherapist), or a specific focus (eg, emotion coaching). Parents whose personal mental health and/or other difficulties hinder them from engaging with and benefitting from the Web-based program will be referred to other mental health services for themselves. Parents who raise significant concerns about their child’s behavior and mental health will also be referred to additional services to better support their child (parents can still continue to use the PIP program if they wish).

## Discussion

### Summary

In this paper, we have described a new approach to developing a Web-based intervention that rigorously translates research evidence into intervention strategies and aligns with more established development models from the parenting program [61] and eHealth intervention [60,62] literature. The PIP intervention is the product of a research translation process to identify the range of potentially modifiable parenting factors for adolescent depression and anxiety [23]. The various components of the intervention were developed to tailor the intervention to each parent’s strengths and areas for improvement, covering the range of factors that are relevant for each family. The intervention can be implemented with varying levels of intervention intensity to meet the level of need of different families at various points along the mental health intervention continuum [2]. PIP is the second intervention developed following this research-translation approach, modeled on the earlier intervention to prevent adolescent alcohol misuse [59]. Such an approach answers the call for better translation of research evidence into interventions [14,15] and can be adopted for other populations (eg, parents of younger children [Fernando LM et al, 2017, unpublished data] and young people [21,77]) and other health and well-being outcomes for which there are a diverse range of risk, protective, and maintenance factors. An important caveat to note about the development process concerns the parent stakeholder consultation group involved in shaping the current version of PIP. Our recruitment for this group used similar methods to those that we predict will underpin the eventual, public implementation of the program, that is, via online networks and through schools. We expect self-selected users of the intervention to have similar characteristics to the parents who comprised our reference groups. To ensure the acceptability of the intervention to underrepresented subgroups of parents (eg, fathers, single parents, and lower income), further consultations with parents from these subgroups would be required [61].

### Challenges and Opportunities for the Implementation Process

An important consideration for the proposed multi-level approach is the source of funding to sustain it. Given that the Web-based intervention is fully developed and evaluated, it is in itself relatively inexpensive to maintain, except when substantial updates and improvements are required. However, where personnel are involved, for either administrative or therapeutic or coaching support, substantial costs will be incurred if the program is implemented at scale. Possible funding models include a user-pays business model, an advertising-based revenue model, or government or third-sector funding. As an international leader in e-mental health [78], Australia is fortunate to have ongoing financial support from the Australian Government for some evidence-based e-mental health programs [79]. As evidence for its efficacy and cost-effectiveness is gathered, such a public health approach may garner the required financial support from the government for its implementation. Moreover, as the program is in a widely understood language such as English, it can potentially be used internationally pending minor cultural adaptations. If this occurs, international funding models will be required [80].

To maximize its uptake and sustainability, the program needs to be integrated into existing public health and health care systems [80]. At a community level, it is important to raise awareness about the program through schools, parenting associations, and other media (including online networks and social media) to facilitate self-referral by parents, or recommendation of the program by teachers, student welfare staff, or school psychologists or counselors to parents within the school. Youth mental health clinicians in the public and private health care systems can refer parents of their youth clients to the fourth level of the program, or deliver it themselves as part their therapeutic work with the adolescent. More broadly, targeted strategies may be required to increase parents’ engagement in parenting programs for their adolescent’s mental health, given that rates of engagement are less than optimal [81]. For harder-to-reach subgroups of parents (eg, parents living in poverty and recent immigrants), additional efforts may be required to improve engagement [82]. Program adaptations may also be needed to make the program more acceptable (and effective) with specific high-risk subgroups, for example, parents of adolescents with autism, disabilities, or chronic health problems.

Research on parent preferences for information on child mental health, in the context of seeking treatment services for their child, also highlights the importance of considering specific preferences of different subgroups [83,84]. For example, a Web-based program will simply not be acceptable to some parents who prefer direct face-to-face contact with a clinician and/or other parents. Similarly, some professionals are skeptical about the ability of Web-based programs to bring about real behavior change and are less likely to recommend it to parents [85]. The parent-preference literature also suggests that parents with the greatest need (ie, higher levels of child oppositional and conduct problems, greater impact of child difficulties on family functioning, and elevated personal depressive symptoms) may be less likely to engage with parenting programs or other resources. Notably, these parents show a stronger preference for information on the Internet, which they can access on demand [83]. These findings highlight the trade-offs between different levels of intervention, which, along with the preferences of various subgroups of parents, should be considered when planning the implementation of parenting programs [86]. For example, parents with the greatest need could just be informed about the availability of the online resources (eg, the Guidelines and the website link) when they first seek mental health services for their child, which is often a time of heightened stress. Once the family settles into treatment and the crisis starts to subside, parents could then be encouraged to consider seeking resources for themselves. Further research on parent preferences for child mental health information for prevention is required.

### Conclusions

Parents have an important role in reducing the risk and impact of adolescent internalizing disorders, but there is a lack of evidence-based, cost-effective programs to equip parents for this role. This paper described the development of the PIP Web-based intervention and proposed a public health approach that utilizes this intervention at varying levels of intensity to support parents. Evaluation of each separate level of the model is ongoing. Further evaluation of the whole approach is required to assess the utility of the intervention as a public health approach, and its effects not just on parenting competencies, parental self-efficacy, and adolescent depression and anxiety outcomes, but also broader functioning (eg, school engagement, general health, quality of life, and peer relationships), and socioeconomic outcomes.

## Appendix

Multimedia Appendix 1 Screenshots of the Partners in Parenting intervention.

Multimedia Appendix 2 Persuasive systems design (PSD) principles fulfilled in the Partners in Parenting (PIP) intervention.

## Abbreviations

CBT: cognitive behavioral therapy
CeHRes: Center for eHealth Research
eHealth: electronic health
PIP: Partners in Parenting
PSD: Persuasive Systems Design
PRADAS: Parenting to Reduce Adolescent Depression and Anxiety Scale
RCT: randomized controlled trial
