# Supplementary material for: Partners in Parenting: A Multi-Level Web-Based Approach to Support Parents in Prevention and Early Intervention for Adolescent Depression and Anxiety
Source: JMIR Ment Health. 2017 Dec 19;4(4):e59. doi: 10.2196/mental.8492 (PMC5750418; doi:10.2196/mental.8492)
Supplement: Multimedia Appendix 1 [file mental_v4i4e59_app1.pdf]

Multimedia Appendix 1. Screenshots of the Partners in Parenting intervention.

(a) Parenting to Reduce Adolescent Depression and Anxiety Scale
(b) Personalised parent feedback
(c) Module selection page
(d) Personalised dashboard
(e) A page of one module

(a)

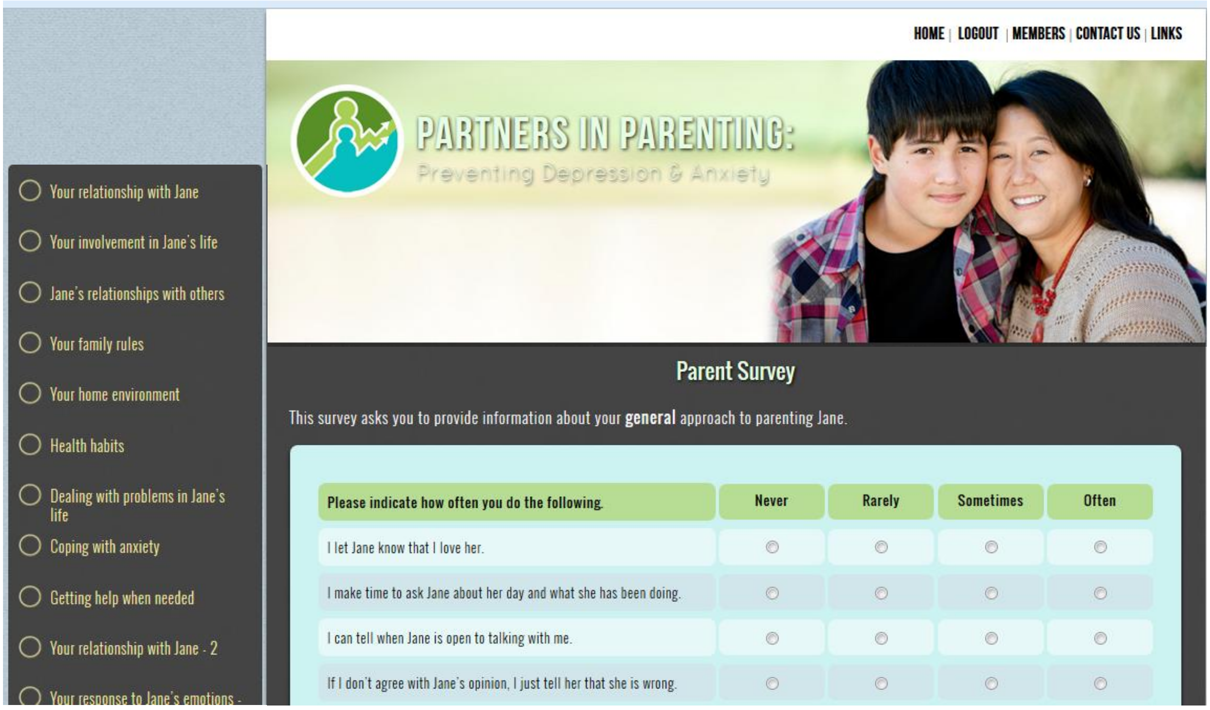

(b)

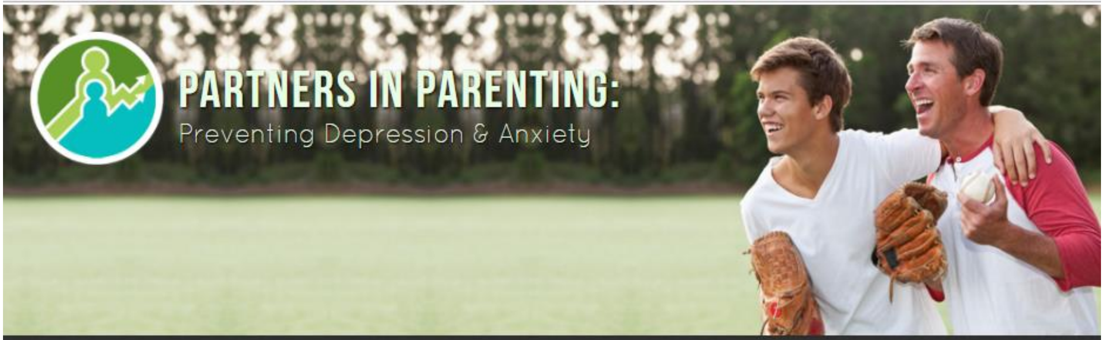

Thank you for completing the survey!

We can now provide you with feedback about your role in reducing Sam's risk of developing depression and clinical anxiety. We also provide you with some practical strategies that you could use to further support Sam. A copy of this feedback has also been sent to your email address.

What next?

This feedback report contains a number of strategies that may be useful for you and Sam. This may seem like a lot of information to take in at once. If you're not sure where to start, you may like to have a quick read over it first. You don't need to try all of the recommended strategies at once. Remember, change can take time and patience. If you feel that there are things you could have done differently as a parent, try not to be too hard on yourself. You've already taken a positive step by completing the survey, well done!

Click on a title to expand your feedback.

Your Relationship with Sam

Your Involvement in Sam's Life

It is important to find a balance between being involved in Sam's life and giving him age-appropriate independence. Teenagers benefit most when their parents continue to show interest in their lives without being intrusive, and respect their need for growing independence.

You can be more involved in Sam's life by:

- Showing an interest in what Sam does at school. For example, you could ask him what he did during the day, what his favourite subjects are, or which teachers he likes.
- If Sam is going out without you, have a casual chat with him about what he will be doing, where he will be, and who he will be with.

You can further support Sam's growing independence by:

- Encouraging Sam to try out extra-curricular activities (e.g. sports, music, or anything else he is interested in).
- Allowing Sam to become more independent of you over time. Evaluate whether you are taking over things too much. For example, you can ask yourself, "Did I really need to step in?" and "What would have been the worst thing to happen if I didn't step in?"
- Gradually increasing Sam's responsibilities and independence over time to allow him to mature.

Sam's Relationships with Others

Your Family Rules

Your Home Environment

Health Habits

Dealing with Problems in Sam's Life

Coping with Anxiety

Getting Help When Needed

(c)

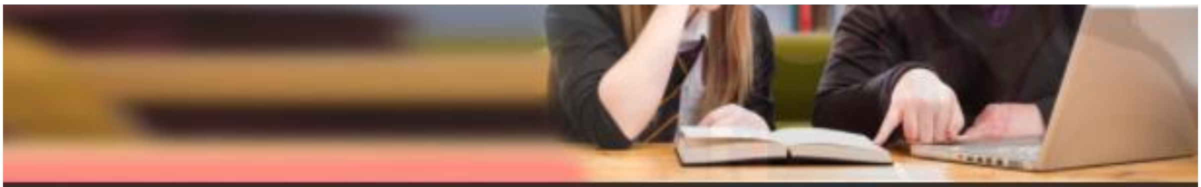

Welcome to your Partners in Parenting program

Based on your responses to the survey, we have recommended specific topics (also referred to as 'modules') in our program just for you (see titles below in orange). These modules correspond to the feedback you have just received, and are designed to support you in making the changes recommended in your feedback messages. However, this is your program, so feel free to choose other topics that are not included in our recommended list.

Each module takes about 15-25 minutes to complete, depending on the topic and the way you choose to engage with it. We encourage you to focus on working through one module a week, to allow yourself time to apply some of the tips and work on your selected goal in the module.

Once you have decided on your modules, click 'Start my program' and you'll be able to check out your first module!

Modules we recommend for you:

- Topic 2: Nurture roots and inspire wings
- Topic 5: Good friends, supportive relationships
- Topic 6: Good health habits
- Topic 7: Partners in problem solving
- Topic 8: From surviving to thriving

Other modules you might want to check out (click to select) :

- Topic 1: Connect
- Topic 3: Raising good kids into great adults
- Topic 4: Calm versus conflict
- Topic 9: When things aren't okay

Start my program

YOUR GOALS

Do you have any goals below to check off as 'completed'?

(d)

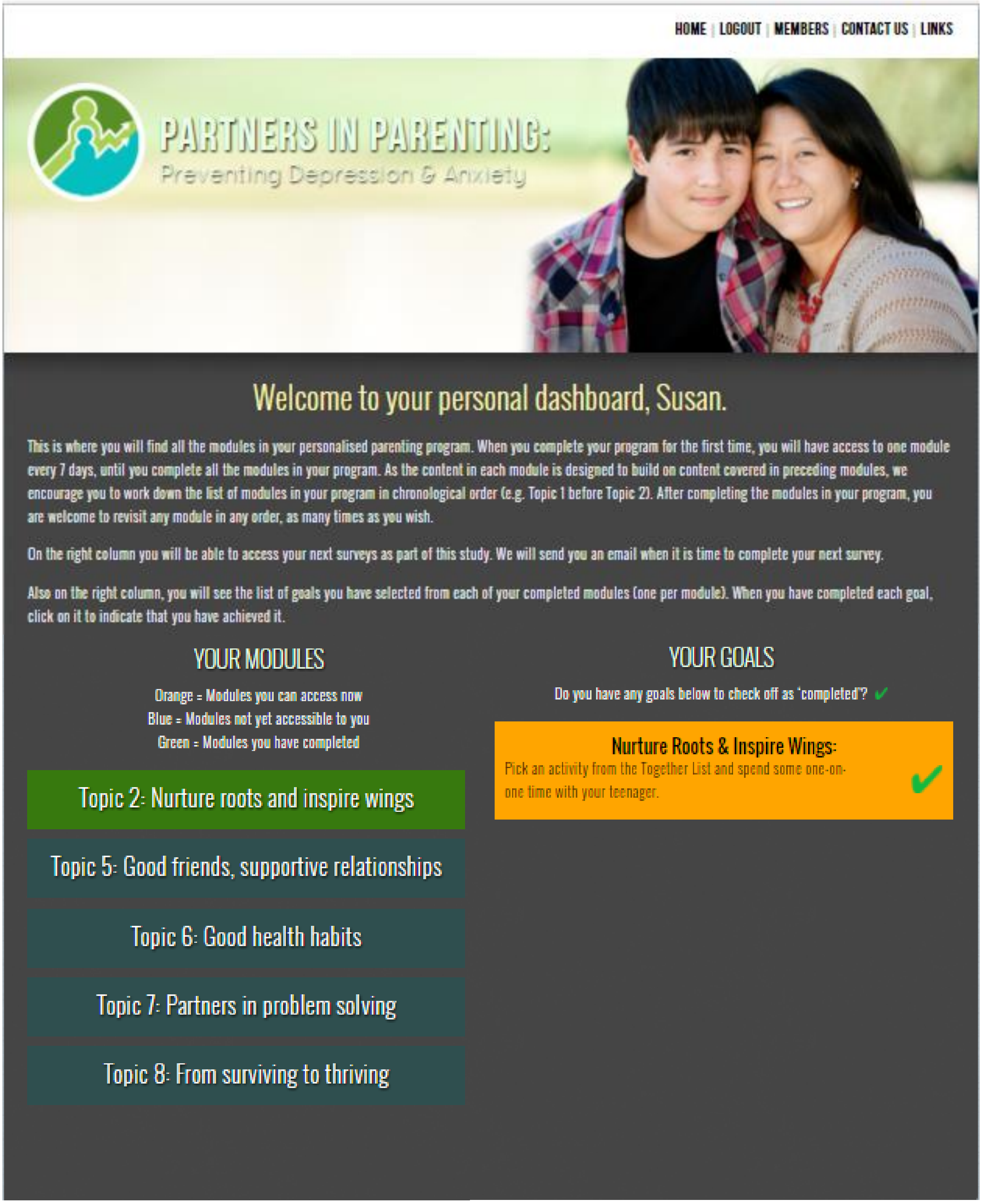

HOME | LOGOUT | MEMBERS | CONTACT US | LINKS
PARTNERS IN PARENTING:
Preventing Depression & Anxiety
Welcome to your personal dashboard, Susan.
This is where you will find all the modules in your personalised parenting program. When you complete your program for the first time, you will have access to one module every 7 days, until you complete all the modules in your program. As the content in each module is designed to build on content covered in preceding modules, we encourage you to work down the list of modules in your program in chronological order (e.g. Topic 1 before Topic 2). After completing the modules in your program, you are welcome to revisit any module in any order, as many times as you wish.
On the right column you will be able to access your next surveys as part of this study. We will send you an email when it is time to complete your next survey.
Also on the right column, you will see the list of goals you have selected from each of your completed modules (one per module). When you have completed each goal, click on it to indicate that you have achieved it.
YOUR MODULES
Orange = Modules you can access now
Blue = Modules not yet accessible to you
Green = Modules you have completed
Topic 2: Nurture roots and inspire wings
Topic 5: Good friends, supportive relationships
Topic 6: Good health habits
Topic 7: Partners in problem solving
Topic 8: From surviving to thriving
YOUR GOALS
Do you have any goals below to check off as 'completed'?
Nurture Roots & Inspire Wings:
Pick an activity from the Together List and spend some one-on-one time with your teenager.

(e)

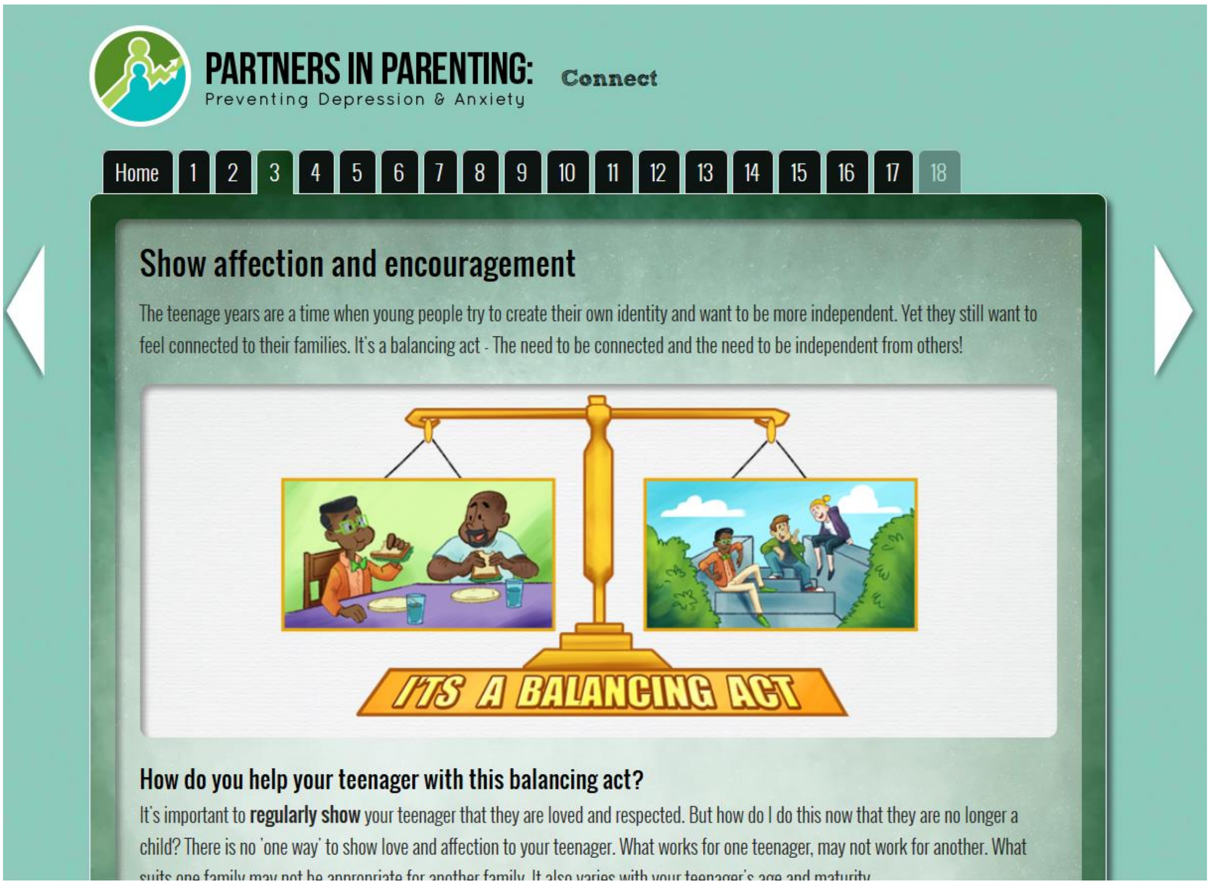

PARTNERS IN PARENTING:
Preventing Depression & Anxiety
Connect
Home 1 2 3 4 5 6 7 8 9 10 11 12 13 14 15 16 17 18
Show affection and encouragement
The teenage years are a time when young people try to create their own identity and want to be more independent. Yet they still want to feel connected to their families. It's a balancing act - The need to be connected and the need to be independent from others!
ITS A BALANCING ACT
How do you help your teenager with this balancing act?
It's important to regularly show your teenager that they are loved and respected. But how do I do this now that they are no longer a child? There is no 'one way' to show love and affection to your teenager. What works for one teenager, may not work for another. What
