# Supplementary material for: Partners in Parenting: A Multi-Level Web-Based Approach to Support Parents in Prevention and Early Intervention for Adolescent Depression and Anxiety
Source: JMIR Ment Health. 2017 Dec 19;4(4):e59. doi: 10.2196/mental.8492 (PMC5750418; doi:10.2196/mental.8492)
Supplement: Multimedia Appendix 2 [file mental_v4i4e59_app2.pdf]

Multimedia Appendix 2: Persuasive systems design (PSD) principles fulfilled in the Partners in Parenting (PIP) intervention.

PSD principles that PIP does not fulfill (rewards and social role from the dialogue support category and all principles in the social support category) are not included here.

PSD[a] principle	Example requirement	Example implementation in PIP[b]
Reduction[c]	Reduce the effort parents need to expend to improve their parenting	Delphi study translates complex research evidence into specific actionable strategies.<br>Tailored feedback reduces a long list of general guidelines to a smaller set of strategies that are relevant for each parent
Tunneling[c]	Guide parents in the attitude change process by providing “means for action” (ie, actionable strategies) that bring them closer to the target behavior	Tailored feedback and module content provide specific strategies parents can use to improve their parenting
Tailoring[c]	Provide information that is tailored to the parent’s potential needs, interests, and other relevant factors	All feedback messages are tailored to each parent.<br>The recommendation of modules from the nine available modules is tailored for each parent
Personalization[c]	Offer personalized content	The Parenting to Reduce Adolescent Depression and Anxiety Scale is personalized for the parent, with each item using the child’s name and gender.<br>Feedback messages are all personalized with the child’s name and gender.<br>Each parent has a personalized dashboard through which they can access their modules.
Self-monitoring[c]	Allow parents to track their progress or status	On their personalized dashboard, parents can see the modules they have accessed and others they have yet to access. They can also monitor their progress through accomplishing each of the goals they had set from their completed modules

Simulation[c]	Provide simulations for parents to observe the link between cause and effect in their behaviors	Modules include vignettes and comic strip illustrations to simulate cause-and-effect links in parenting behaviors
Rehearsal[c]	Provide means for parents to rehearse target behaviors	Modules have interactive activities requiring parents to rehearse target behaviors
Praise[d]	Provide praise (eg, words and images) as a form of positive reinforcement for parent behaviors	Feedback messages praise parents for their areas of strength.<br>Automated emails praise and congratulate parents upon module and goal completion
Reminders[d]	Remind parents of their target behaviors during their use of the system	Parents are reminded of their goals via email and on their personalized dashboard
Suggestion[d]	Suggest specific behaviors that parents can carry out	Feedback messages and module content include many specific actions that parents can take
Similarity[d]	Have features that remind parents of themselves in some meaningful way	Illustrations, vignettes, quotes, and adolescent audio clips presenting a wide variety of possible parent-child interactions are used to connect parents to the content in a meaningful way
Liking[d]	Have a look and feel that parents find appealing and attractive	Full color illustrations, clean cut website design, and a moderate level of dynamic, interactive features are used based on parent reference group feedback
Trustworthiness[e]	Provide information that is truthful, fair, and unbiased	All components of the intervention present evidence-based and/or expert-endorsed information in a conversational but matter-of-factly, professional tone
Expertise[e]	Provide information showing knowledge, experience, and competence	Parents are informed that the intervention is developed by researchers at renowned universities based on guidelines that were developed in partnership with *beyondblue*, a respected mental health organization
Surface credibility[e]	Have a competent look and feel	The website has a clean cut, uncluttered design (eg, no

		advertisements) for easy navigation
Real-world feel[e]	Provide information of the organization(s) and actual people behind the intervention content	Parents are informed about the researchers and organizations behind the intervention.<br>The website provides email and phone contact details that parents can use for enquiries and feedback
Authority[e]	Refer to people in the role of authority	The guidelines, feedback messages, and modules cite published research evidence and other authorities (eg, government guidelines)
Third-party endorsements[e]	Provide endorsements from respected sources	The website has a medal logo showing that the program has been awarded the Australian Rotary Health Knowledge Dissemination Award for "excellence in research translation"
Verifiability[e]	Provide parents with ways to verify the accuracy of intervention content with outside sources	External sources that are cited in all components of the intervention have accompanying bibliographic information that parents can look up for verification

[a]PSD: persuasive systems design.

[b]PIP: Partners in Parenting.

[c]Principles from the primary task support category.

[d]Principles from the dialogue support category.

[e]Principles from the system credibility support category.
